# Glucose-Regulated Protein 78 in the Aqueous Humor of Patients with Diabetic Macular Edema

**DOI:** 10.1155/2020/1640162

**Published:** 2020-02-23

**Authors:** Moon Young Choi, Jin-woo Kwon

**Affiliations:** Department of Ophthalmology, St. Vincent's Hospital, College of Medicine, Catholic University of Korea, Republic of Korea

## Abstract

**Purpose:**

We identified the associations between levels of aqueous glucose-regulated protein 78 (GRP78) and systemic or ocular factors in patients with center-involving diabetic macular edema (CIDME).

**Methods:**

We measured the aqueous concentrations of GRP78, interleukin- (IL-) 1*β*, IL-2, IL-8, IL-10, and IL-17, placental growth factor, and vascular endothelial growth factor (VEGF). We explored the associations between aqueous GRP78 levels and those of other aqueous factors, optical coherence tomography (OCT) findings, and systemic parameters in CIDME patients.

**Results:**

In multivariate regression analysis, aqueous GRP78 levels were associated with aqueous VEGF levels (*p* = 0.007), length of EZ disruption (*p* = 0.007), length of EZ disruption (*p* = 0.007), length of EZ disruption (*p* = 0.007), length of EZ disruption (

**Conclusions:**

Aqueous GRP78 levels correlated with VEGF levels in the aqueous humor and EZ disruption on OCT. However, GRP78 levels were not associated with those of inflammatory biomarkers in the aqueous humor or OCT findings. Additionally, GRP78 could not serve as a biomarker to predict short-term prognosis of anti-VEGF agent.

## 1. Introduction

The endoplasmic reticulum (ER) exhibits several metabolic and biosynthetic functions. During protein synthesis, unfolded and misfolded proteins can accumulate in the ER; ER stress is defined as abnormally excessive accumulation of these proteins in the organelle under pathological conditions [1]. As the unfolded protein response is then initiated, the chaperone proteins are activated to complete protein folding or to disassemble abnormal proteins [2, 3]. If there is no proper chaperone activity, ER stress may trigger apoptosis or autophagy [4, 5]. Glucose-regulated protein 78 (GRP78), a representative ER chaperone, has served as ER stress marker [6, 7]. GRP78 facilitates appropriate protein processing, degrades misfolded proteins, ensures calcium homeostasis, and activates transmembrane ER stress sensor proteins [8–10]. Inflammation and ischemia are the most common pathological conditions that trigger ER stress [11, 12]. Many reports have shown that ER stress is associated with diabetes [13–15]. Additionally, the early pathogenesis of diabetic macular edema (DME) usually involves damage to the inner blood-retinal barrier caused by hypoxia and inflammation [16]. However, few studies have explored the relationship between DME and ER stress [17]. Thus, we designed this study to identify the factors that affect their relationship by exploring GRP78 levels in the aqueous humor of DME patients.

## 2. Methods

### 2.1. Study Subjects

We assessed the association of GRP78 levels, measured in the aqueous humor, with levels of interleukin- (IL-) 1*β*, IL-2, IL-8, IL-10, and IL-17; placental growth factor (PlGF); and vascular endothelial growth factor (VEGF), as well as with the optical coherence tomography (OCT) findings of center-involving DME (CIDME) with type II diabetes mellitus (DM) [18, 19]. This prospective study adhered to all relevant tenets of the Declaration of Helsinki, and the protocol was approved by the institutional review ethics board of the Catholic University of Korea. All participants provided written informed consent for use of their clinical records.

We enrolled treatment-naïve CIDME eyes of central subfield thickness (CST) ≥ 300 *μ*m from 2016 to 2018 [18, 19]. The exclusion criteria included macular edema attributable to other causes. We also excluded eyes with concurrent ocular conditions such as glaucoma, high myopia with axial length ≥ 25.5 mm, and retinal vascular occlusion and eyes with histories of prior ocular conditions including uveitis and intraocular surgery or laser that could influence enzyme levels in the aqueous humor. We measured glycated hemoglobin (HbA1c) levels in all patients and performed complete ophthalmic examinations, including measurement of best-corrected visual acuity (BCVA), fundus examination, and fluorescein angiography (FAG) to classify the eyes according to the Early Treatment of Diabetic Retinopathy Study as mild nonproliferative diabetic retinopathy (NPDR), moderate and severe NPDR, or proliferative diabetic retinopathy (PDR). We also assessed CST and macular morphology via OCT (Cirrus High-Definition OCT; Carl Zeiss Meditec, Dublin, CA, USA), and the axial length was measured using an IOL Master instrument (Carl Zeiss Meditec). We used a horizontal B-scan OCT image centered on the fovea for measuring hyperreflective foci (HF) and total length of ellipsoid zone (EZ) disruption within the central 3000 *μ*m around the fovea [20, 21]. We evaluated changes in CST at the 1-month visit after three consecutive monthly intravitreal bevacizumab (IVB) injections. Responsiveness was defined as CST < 300 *μ*m after these treatments.

### 2.2. Quantitation of GRP78 in the Aqueous Humor

GRP78 protein levels were determined in 1 : 1 diluted aqueous humor samples using a human GRP78 enzyme-linked immunosorbent assay (ELISA) kit (Enzo Life Sciences, Lausen, Switzerland) with a detection range of 1.4 to 4500 ng/mL according to the manufacturer's instructions. All values under the lower limit of detection were considered zero values.

### 2.3. Assay of Cytokines and Growth Factors

We assayed IL-1*β*, IL-2, IL-8, IL-10, IL-17, PlGF, and VEGF levels in the aqueous humor from the anterior chamber using bead-immobilized human antibodies against these materials; 75 *μ*L humor samples and 75 *μ*L amounts of calibrator diluent RD6-52 were added to the beads, incubated for 2 h at room temperature (20–25°C), and then for a further 1 h at room temperature after the addition of detection antibodies, and for a final 30 min at room temperature after the addition of the streptavidin-phycoerythrin reagent. A Luminex xMAP suspension array system (Luminex, Austin, TX, USA) was used for detection; this is a multiplexed microsphere suspension immunoassay that detects and quantitates spectrally unique microspheres attached to specific antibodies. The detection limits and dynamic ranges are as follows: 0.8 pg/mL with a dynamic range to 3950 pg/mL for IL-1*β*, 1.8 pg/mL with a dynamic range to 1140 pg/mL for IL-8, 1.6 pg/mL with a dynamic range to 890 pg/mL for IL-10, 1.8 pg/mL with a dynamic range to 2090 pg/mL for IL-17, 1.9 pg/mL with a dynamic range to 470 pg/mL for PlGF, and 2.1 pg/mL with a dynamic range to 2170 pg/mL for VEGF. All values under the lower limit of detection were considered zero values [22, 23].

### 2.4. Statistical Analyses

We performed between-group comparisons using Student's *t*-test, the Mann-Whitney *U* test, and the chi-squared test. Linear regression analyses were employed to identify factors associated with GRP78 levels, and Spearman's rank correlation was used for univariate analysis. All statistical analyses were performed using SPSS for Windows software (ver. 21.0; SPSS Inc., Chicago, IL, USA) and R software (ver. 3.2.3; R Development Core Team, 2015).

## 3. Results

We enrolled 66 treatment-naïve CIDME eyes of 66 patients with a mean age of 56.70 ± 8.07 years; there were 28 males and 38 females. Forty-nine (74.24%) patients had proliferative DR and 17 (25.76%) had nonproliferative DR. The mean BCVA (logMAR) was 0.49 ± 0.24, and the mean CST was 414.53 ± 79.44 *μ*m at baseline. In the classification by DME morphology based on the OCT, 30 patients had cystoid macular edema and 36 had diffuse retinal thickening (Table 1).

In univariate regression analysis, GRP78 levels in the aqueous humor were associated with aqueous VEGF levels (*p* = 0.032), length of EZ disruption (*p* < 0.001), and number of HF (*p* = 0.006). In multivariate regression analysis, GRP78 levels in the aqueous humor were associated with aqueous VEGF levels (*p* = 0.007), length of EZ disruption (*p* < 0.001), and duration of diabetes (*p* = 0.002) (Table 2).

Of the 66 ME patients, 23 (34.85%) exhibited CST < 300 *μ*m 1 month after the last IVB injection. Multivariate logistic regression analysis showed that HF ≥ 8 on OCT finding was the only factor associated with responsiveness (OR = 0.34, *p* = 0.046), but aqueous GRP78 was not associated with the responsiveness (Table 3).

## 4. Discussion

GRP78 plays multiple roles in the ER, serving mainly as a chaperone [8, 9]. All chaperones have a lysine-aspartate-glutamate-leucine (KDEL) ER retention signal [24]. Under excessive stress on the ER, the KDEL receptor expression cannot be coordinately upregulated, and as a result, KDEL receptors become saturated [25]. GRP78 can escape from the ER and appear on the cell surface or be out of the cell [26]. Surface GRP78 mediates cell proliferation, apoptosis, and immune activity [27, 28]. On the other hand, the action of secreted form of GRP78 is not as well-known as those of the other forms. As diabetic retinopathy develops under ER stress conditions [29–31], we explored the association between secreted GRP78 levels in the aqueous humor and DME development and found that GRP78 levels were associated with those of aqueous VEGF and may reflect retinal status.

As ER stress develops during ischemia or inflammation, DME is also associated with these conditions. The mainstay of DME treatment is injection of intravitreal anti-VEGF or steroid; an agent is selected by reference to prior responsiveness to that agent or as suggested by FAG or OCT [32, 33]. Several studies have sought biomarkers that predict treatment responsiveness and prognosis [34, 35]. Previously, we had reported that the aqueous GRP78 levels had a correlation with VEGF levels in the aqueous humor, but the report had limitation; it could not analyze the association between aqueous GRP78 levels and inflammation [17]. In this study, aqueous GRP78 levels showed no significant correlations with proinflammatory or anti-inflammatory biomarkers in the aqueous humor.

We aimed to determine the mechanism involved in DME associated with ER stress and whether GRP78 could serve as a useful biomarker.

On multivariate regression analysis, aqueous GRP78 levels were significantly correlated with EZ disruption grades but not with HF numbers on OCT. Previously, HF were suspected as subclinical features of lipoprotein extravasation or migrating pieces of retinal pigmented epithelium [36, 37]. However, recent reports suggested that HF can be activated forms of microglia and markers of inflammation [38, 39]. On the other hand, EZ integrity reflects the health of the photoreceptor layer; EZ disruption is associated with a poor visual prognosis in DME patients [40]. Some studies have already shown that EZ disruption may also be associated with ischemia. One reported foveal ischemia in DME causes EZ disruption resulting in outer retinal layer changes [41], and another study suggested that macular ischemia in diabetes may be associated with photoreceptor compromise and ischemia at the deep capillary plexus may play an important role in these outer retinal changes [41]. Our OCT findings may indicate that GRP78 levels reflect retinal ischemic status.

There have been some studies demonstrating associations of HF in DME and anti-VEGF responsiveness. Similar to our results, they have reported that higher number of HF could be a poor prognostic factor result in worse BCVA or less CST reduction after anti-VEGF treatments in DME patients [42, 43]. Recent study reported that intravitreal dexamethasone implant treatment was superior to anti-VEGF agents in DME with serous retinal detachment and HF [44].

To sum up, aqueous VEGF levels and EZ disruption on OCT were associated with aqueous GRP78 levels, but the levels of inflammatory markers of the various IL families in the aqueous humor and HF as inflammatory biomarkers in OCT were not. Considering these results together, the data indicate that GRP78 levels seem to be more closely associated with ischemia than with inflammation.

This study had certain limitations. First, the sample size and volume were too small to explore how GRP78 levels affect functional and anatomical parameters, and aqueous volume was not enough for duplication in the ELISA analysis. Second, any interaction between GRP78 and VEGF remains unclear. Although some studies have reported that GRP78 levels correlated both with the extent of neovascularization and elevated VEGF levels [45–47], the role of GRP78 in DME pathogenesis cannot be definitively proven by their detection in aqueous samples. Third, when exploring retinal ischemia, OCT angiography would have been helpful. Despite these limitations, this is the first study to show that aqueous GRP78 levels correlate with both VEGF levels in the aqueous humor and EZ disruption revealed by OCT, implying that GRP78 levels may reflect retinal ischemic status in DME patients. Future studies should explore the role and origin of this ER chaperone in DME.

## Figures and Tables

**Table 1:** Demographics and clinical characteristics of the center-involving diabetic macular edema patients.

		*N* = 66
Systemic factors	Sex (male : female)	28 : 38
	Age (years)	56.70 ± 8.07
	HbA1C (%)	7.59 ± 0.94
	Duration of DM (years)	13.00 [7.00; 18.00]

OCT findings	Number of HF	8.00 [6.00; 13.00]
	Subretinal fluid	12 (18.18%)
	CME : DRT	30 : 36
	EZ disruption	
	(-)	42 (63.64%)
	(+)	24 (36.36%)

Aqueous humor components	GRP78 (ng/mL)	4.05 [2.64; 5.22]
	IL-1*β* (pg/mL)	3.49 [1.86; 3.49]
	IL-8 (pg/mL)	18.84 [11.62; 28.49]
	IL-10 (pg/mL)	0.00 [0.00; 0.00]
	IL-17 (pg/mL)	2.56 [0.00; 2.76]
	PlGF (pg/mL)	0.00 [0.00; 2.23]
	VEGF (pg/mL)	65.22 [31.65; 95.58]

Ocular factors	Baseline BCVA (logMAR)	0.50 [0.40; 0.70]
	Baseline CST (*μ*m)	391.00 [363.00; 457.00]
	DMR (NPDR : PDR)	17 : 49

Values are expressed as means ± SDs or as medians with interquartile ranges, as appropriate. HbA1c: glycated hemoglobin; OCT: optical coherence tomography; HF: hyperreflective foci; CME: cystoid macular edema; DRT: diffuse retinal thickening; EZ: ellipsoid zone; GRP78: glucose-regulated protein of 78 kDa; IL: interleukin; PlGF: placental growth factor; VEGF: vascular endothelial growth factor; BCVA: best-corrected visual acuity; CST: central subfield thickness; DMR: DM retinopathy; NPDR: nonproliferative diabetic retinopathy; PDR: proliferative diabetic retinopathy.

**Table 2:** Parameters associated with the glucose-regulated protein of 78 kDa (GRP78) levels in the aqueous humor of center-involving diabetic macular edema patients in univariate and multivariate regression analyses.

		Univariate^∗^		Stepwise backward^∗^^†^	
		*B* ± SE	*p* value	*B* ± SE	*p* value
Systemic factors	HbA1c (%)	0.58 ± 0.36	0.112		
	Duration of diabetes (years)	0.07 ± 0.05	0.107	0.12 ± 0.04	0.002

OCT findings	Preoperative CST (*μ*m)	0.00 ± 0.00	0.828		
	Length of EZ disruption (*μ*m)	0.01 ± 0.00	<0.001	0.01 ± 0.00	<0.001
	Number of HF	0.18 ± 0.06	0.006	0.11 ± 0.06	0.059

Aqueous humor components	IL-1*β* level (pg/mL)	−0.04 ± 0.21	0.855		
	IL-8 level (pg/mL)	0.02 ± 0.02	0.3584		
	IL-10 level (pg/mL)	−0.03 ± 0.54	0.953		
	IL-17 level (pg/mL)	0.19 ± 0.18	0.277		
	PlGF level (pg/mL)	0.04 ± 0.12	0.738		
	VEGF level (pg/mL)	0.01 ± 0.01	0.032	0.01 ± 0.00	0.007

*B*: estimated regression coefficient; HbA1c: glycated hemoglobin; OCT: optical coherence tomography; CST: central subfield thickness; EZ: ellipsoid zone; HF: hyperreflective foci; IL: interleukin; PlGF: placental growth factor; VEGF: vascular endothelial growth factor. ^∗^Adjusted for age, sex, and diabetic retinopathy stage. ^†^Adjusted *R*^2^ = 0.439.

**Table 3:** Results of logistic regression, effect on responsiveness to intravitreal bevacizumab treatments.

	Category	*n* (%)	Univariate		Multivariate	
			OR (95% CI)	*p*	OR (95% CI)	*p*
Sex	Female	38 (57.58%)	Reference			
	Male	28 (42.42%)	0.61 (0.21, 1.72)	0.360		

Age (years)	<60	30 (45.45%)	Reference			
	≥60	36 (54.55%)	0.84 (0.33, 1.27)	0.558		

HbA1c	≤7	18 (%)	Reference			
	>7	48 (%)	1.56 (0.50, 5.52)	0.462		

DMR stage	NPDR	17 (27.27%)	Reference			
	PDR	49 (72.73%)	0.69 (0.22, 2.22)	0.526		

EZ disruption	(-)	42 (63.64%)	Reference			
	(+)	24 (36.36%)	0.67 (0.22, 1.93)	0.465		

Number of HF	<8	29 (43.94%)	Reference		Reference	
	≥8	37 (56.06%)	0.34 (0.12, 0.96)	0.046	0.34 (0.12, 0.96)	0.046

GRP78 (ng/mL)	>4.051	33 (50.00%)	Reference			
	<4.051	33 (50.00%)	0.88 (0.31, 2.42)	0.796		

OR: odds ratio; CI: confidence interval; HbA1c: glycated hemoglobin; DMR: DM retinopathy; EZ: ellipsoid zone; HF: hyperreflective foci; GRP78: glucose-regulated protein of 78 kDa.

## Data Availability

The datasets generated during and/or analyzed during the current study are available from the corresponding author on reasonable request.
